# Clinical Spectrum and Causes of Delayed Puberty Among Patients Presenting to the Endocrine Clinic at Jinnah Postgraduate Medical Centre

**DOI:** 10.7759/cureus.21574

**Published:** 2022-01-24

**Authors:** Fatima Zahra, Tasnim Ahsan, Urooj Lal Rehman, Rakhshanda Jabeen

**Affiliations:** 1 Medicine, Fazaia Ruth Pfau Medical College (FRPMC), Karachi, PAK; 2 Diabetes and Endocrinology, Medicell Institute of Diabetes Endocrinology & Metabolism, Karachi, PAK; 3 Medicine, Jinnah Postgraduate Medical Centre, Karachi, PAK; 4 Internal Medicine, Jinnah Postgraduate Medical Centre, Karachi, PAK

**Keywords:** delayed puberty, constitutional delay, hypogonadotropic hypogonadism, hypergonadotropic hypogonadism, short stature, amenorrhea, turner syndrome, klinefelter syndrome

## Abstract

Introduction: It has been observed that 5% of adolescents are affected by pubertal timing disorders. However, there is limited data about this in Pakistan. This cross-sectional study aimed to observe the patterns and causes of delayed puberty (DP) among patients presenting at the endocrine clinic of a tertiary care hospital in Karachi.

Methods: This observational study was conducted at the endocrine clinic of Jinnah Postgraduate Medical Centre (JPMC) Unit II from 2007 to 2015. A detailed history was obtained from patients presenting with DP. We noted the available demographic data, main complaints, and family history of DP. Physical examinations were performed and the data recorded. Tanner staging was used to assess pubertal development. Relevant laboratory and imaging investigations were performed; data analysis was performed using SPSS 17 (IBM Corp., Armonk, NY).

Results: A total of 2670 patients were registered in the endocrine clinic during the study period, of which 171 presented with DP; 119 were males and 52 were females. There was a wide variation in age at presentation ranging from 10 to 32 years. The majority of patients presented with short stature - 69 (57.98%) males and 19 (36.53%) females. Small testes were present in 28 patients (23.52%); 19 (15.96%) males presented with absent secondary sexual characteristics and infertility was present in three (2.54%) males, primary amenorrhea was observed in 25 (48.07%), both primary amenorrhea and short stature were the presenting symptoms of five (9.61%), and failure of breast development was seen in three (5.76%) females. Constitutional delayed growth and puberty (CDGP) was diagnosed in 42 patients (24.6%). The definitive diagnosis of idiopathic hypogonadotropic hypogonadism (IHH) was made in 18 (10.5%) patients. In another 18 (10.5%) patients, we could not differentiate between CDGP and IHH. Functional hypogonadotropic hypogonadism (FHH) due to non-endocrine illness was present in 16 patients (9.4%). The cause of DP was hypogonadotropic hypogonadism in 33 (19.3%) patients whereas 44 patients presenting with DP could not be classified due to incomplete data.

Conclusion: This study showed that CDGP was the most common cause of DP in our patients with the most common presentation being short stature in males and amenorrhea in females. It is essential to differentiate CDGP in children from a small fraction of the pathological and treatable causes of DP.

## Introduction

Delayed puberty (DP) is observed in 5% of adolescents and can impair health and psychosocial outcomes [1-3]. One review quoted that approximately 3% of children may have variations in normal puberty [4]. Usually, more boys seek medical attention for DP than girls because of their concern about short stature compared to their peers [5]. DP is observed in approximately two-thirds of cases due to constitutional delay [6]. Pubertal delay can also be the result of chronic illness, hypogonadotropic or hypogonadotropic hypogonadism, or both [7]. Distinguishing idiopathic hypogonadotropic hypogonadism (IHH) from constitutional delay is an important clinical issue for initiating treatment, which may require prolonged follow-up. A local study also reported a dilemma in differentiating these two conditions [8].

Evaluation and understanding of DP is a neglected area of endocrinology for both researchers and healthcare providers. Although some research has been conducted, it tends to focus on relatively small groups and often involves a single gender; therefore, data about the presentation and causes of delayed puberty are insufficient [9,10]. The aim of this study was to determine the causes and different modes of presentation of delayed puberty in both sexes.

## Materials and methods

This observational study was a retrospective analysis of the patients who presented at the endocrine clinic of Jinnah Postgraduate Medical Centre (JPMC) Unit II from 2007 to 2015. Approval of the institutional ethical review committee of the JPMC was obtained prior to enrollment of subjects. A non-probability convenience sampling technique was used. The selection criteria of patients included the recorded history of all patients presenting with any of the following: a) short stature (both sexes) b) amenorrhea/absent breast development (females), c) absent testicular enlargement (males) and d) absent secondary sexual characteristics (both). We noted the available demographic data, main complaints, and family history of delayed puberty in a predesigned questionnaire.

Physical examination was recorded from the maintained data, including height and weight, to calculate body mass index. Tanner staging was used to assess pubertal development. In males, testicular size was measured using a standard orchidometer. Complete blood count, iron studies (performed in patients with anemia), and anti-tissue transglutaminase antibodies (for coeliac disease in anemic patients) were recorded. Ultrasound pelvis was done in females to assess the ovaries and uterus. Basal hormonal studies included luteinizing hormone, follicle-stimulating hormone, testosterone, estradiol, and thyroid hormones. Bone age was determined by radiography of the nondominant wrist, using the standards of Greulich and Pyle’s Atlas of Skeletal Development, and MRI of the pituitary was performed in patients who had hypogonadotropic hypogonadism, whereas chromosomal analysis was reserved for patients who had hypogonadotropic hypogonadism. Data were analyzed using SPSS 17 (IBM Corp., Armonk, NY). Frequencies and percentages were determined for categorical data, whereas mean, median, and standard deviations were calculated for continuous data.

## Results

A total of 171 patients were enrolled in the study for delayed puberty in the endocrine clinic of JPMC where 119 were males and 52 were females. The mean age was 17.43±2 years. The complaints for which they presented at the endocrine OPD are shown in Table 1 for both males and females; the highest frequency is short stature.

**Table 1:** Presenting Complaints of Patients with Delayed Puberty

Presenting Complaints	Males (119) n(%)	Females (52) n(%)	p value*
Short stature	69 (57.98)	19 (36.53)	0.01
Small testes	28 (23.52)	---	
Absent secondary sexual characteristics	19 (15.96)	---	
Primary infertility and absent secondary sexual characteristics	3 (2.54)	---	
Primary amenorrhea	---	25 (48.07)	
Primary amenorrhea and short stature	---	5 (9.61)	
Absent breast enlargement	---	3 (5.76)	

On the basis of hormonal analysis, they were grouped into hypogonadotropic hypogonadism, 94 (54.97%) patients; hypogonadotropic hypogonadism, 33 (19.3%) patients with a significant difference in gender (p=0.019), and 44 (25.7%) patients who were not diagnosed appropriately. The patients with hypogonadotropic hypogonadism were further divided into four categories: constitutional delayed growth and puberty (CDGP) in 24.6% patients, CDGP/IHH in 10.5% patients, IHH in 10.5% patients, functional hypogonadotropic hypogonadism (FHH) in 9.4% patients with no significant difference in both genders as shown in Table 2. IHH was diagnosed if endogenous puberty had not begun by the age of 16 years in females and 18 years in males. The males who presented below 18 years and had testicular size >4 ml, were categorized as CDGP, whereas those who had testicular size <4 ml could not be categorized into CDGP or IHH without prolonged follow-up for spontaneous puberty or further investigations

**Table 2:** Frequency of Different Categories of Diagnosis

Different categories of diagnosis	Number of patients (171) n(%)	Males (119) n(%)	Females (52) n(%)	p value*
Unknown etiology	44(25.7)	33(27.7)	11(21.2)	0.365
Constitutional delay of growth and puberty (CDGP)	42(24.6)	26 (21.8)	16(30.8)	0.213
Hypergonadotropic hypogonadism	33(19.3)	18(15.1)	15(28.9)	0.019
Idiopathic hypogonadotropic hypogonadism (IHH)	18(10.5)	12(10.1)	6(11.5)	0.776
Constitutional delay of growth and puberty/IHH	18 (10.5)	18(15.1)	---	---
Functional hypogonadotropic hypogonadism	16(9.4)	12(10.1)	4(7.7)	0.621

The causes of FHH among our male patients were as follows: celiac disease was diagnosed in four boys; hypothyroidism, hyperthyroidism and subclinical hyperthyroidism each one was observed in one patient. Cranial diseases included pituitary lesions which were non-secretory adenomas in two patients and prolactinoma in one patient. Among females, celiac disease and hypothyroidism were diagnosed in one patient each. Karyotyping was advised to all patients with hypogonadotropic hypogonadism and the results are shown in Table 3.

**Table 3:** Karyotyping Results of Hypogonadotropic Hypogonadism

Karyotyping	n = 33	Percent
Klinefelter's syndrome XXY	5	15.2 %
Turners syndrome XO	9	27.3 %
Normal	8	24.2 %
Not done	11	33.3 %

## Discussion

We observed that 6.4% of all patients presented with DP in our endocrine clinic but the prevalence of delayed puberty cannot be estimated in our population due to a lack of local data. In this study, DP was observed in a greater number of males (69.5%) than in females (30.4%). The male predominance observed in DP may be due to gender bias in society [11].

In our study, the mean age of patients was 17+2 years indicating they may delay seeking medical advice. The age at puberty onset depends on the racial origin and genotype. Hagen et al. pointed out in their study that Asian girls reach puberty later and earlier puberty was observed in women of African origin as compared to Europeans. They also found that breast development age for patients with delayed puberty was 13.8 years (13.0-17.0) [12]. Individuals with DP have an age of pubertal onset outside of the statistical definition of normal pubertal timing, with the absence of testicular enlargement in boys, and breast development in girls at an age that is 2-2.5 standard deviations later than the population mean [13].

The results of our study showed short stature as the most common presentation of DP in males while females have primary amenorrhea as the lead presentation. Many patients with DP have growth failure of the breast or testes or absence of secondary sexual characteristics such as axillary or genital hair growth or changes in voice and appearance of mustaches and beards in males. The prevalence of short stature in both sexes is affected by ethnicity, heredity, and environmental factors. Genetic variation in healthy individuals can also lead to short stature (about 90% of cases), but at times, it can reflect, undiagnosed diseases such as celiac disease, inflammatory bowel disease, endocrine disorders, and psychosocial deprivation [14].

In gynecological clinics, primary amenorrhea is the most common presentation among adolescent girls [15]. It can be caused by outflow obstruction or endocrine diseases of the pituitary or thyroid adrenal.

The most common cause of DP worldwide is CDGP, which was also reflected in this study. We found CDGP in 24.6% of the patients with DP. In a local study, it was observed that the cause of primary amenorrhea was CDGP in 10.52% of females [9]. According to the analysis of a large case series by Sedlmeyer and Palmert., CDGP affected 53% of the subjects (63% of males and 30% of females) [5]. The influence of gender, ethnicity, and study population contributes to the disparity in numbers. We observed overlapping of CDGP and IHH, as it was extremely difficult to differentiate two with certainty in 10.5% of males [10].The incidence of IHH is 1-10 cases per 100,000 births [16]. We observed that 10.5% of patients had IHH, 9.4% had FHH, and 19.3% had hypogonadotropic hypogonadism. Comparable results can be also observed in the Sedlmeyer study, which reported FHH in 19% of individuals; IHH in 12% of patients and 13% of patients had hypogonadotropic hypogonadism.

The FHH group included patients with thyroid disease, including hypothyroidism, hyperthyroidism, and subclinical hyperthyroidism. Interestingly, three males and one female with thyroid disorders presented with DP. Thyroid dysfunction can lead to delayed puberty by increasing the gonadotropin inhibitory hormone [17]. There are CNS disorders such as pituitary nonsecretory adenoma and prolactinoma; the rare cause of arrested puberty in the pediatric population should be considered in children for earlier intervention [18].

Among hypogonadotropic hypogonadism, Kallmann Syndrome (KS) was the most common cause of DP (15.2%). We observed that the mean age of patients with KS was 25 years. KS has been reported to be severely underdiagnosed or diagnosed late in life, usually on workup for infertility; roughly 25% are diagnosed, and the mean age of diagnosis is in the mid-30s [19]. Bojesen also found that 65% to 85% of KS patients consulted doctors after the age of 25 years for overt hypogonadism signs [20]. Early detection of KS is highly recommended to initiate treatment for hypogonadism prevent osteoporosis, and improve psychosocial problems.

We diagnosed Turner syndrome in 27.3% of females who had primary amenorrhea.TS is a common sex chromosome abnormality in women, with an incidence of one in 2500 female births [21]. The diagnosis of TS and KS would be missed less often if doctors and patients were more aware of key manifestations, as it may cause infertility issues later in life [22].

A limitation of our study is that we observed different presentations of DP but could not classify 25.7% of our patients in an appropriate group due to limited investigations in a government facility although a local study (about short stature only) also reported 2.4% unknown syndrome [23]. Further research is needed to establish appropriate age cutoffs for DP in different racial and ethnic groups and to better understand the physiological basis of CDGP. National guidelines are needed regarding delayed puberty and its management options in appropriate candidates.

## Conclusions

This study showed that CDGP was the most common cause of DP in our patients with the most common presentation being short stature in males and amenorrhea in females. It is essential to differentiate CDGP in children from a small fraction of the pathological and treatable causes of delayed puberty.
